# Hawthorn fruit extract reduced trimethylamine-*N*-oxide (TMAO)-exacerbated atherogenesis in mice via anti-inflammation and anti-oxidation

**DOI:** 10.1186/s12986-020-00535-y

**Published:** 2021-01-07

**Authors:** Zouyan He, Erika Kwek, Wangjun Hao, Hanyue Zhu, Jianhui Liu, Ka Ying Ma, Zhen-Yu Chen

**Affiliations:** 1 School of Life Sciences, The Chinese University of Hong Kong, Shatin, Hong Kong China; 2 School of Public Health, Guangxi Medical University, Nanning, 530021 China; 3 College of Food Science and Engineering, Nanjing University of Finance and Economics, Nanjing, 210023 China

**Keywords:** Trimethylamine-*N*-oxide, Atherosclerosis, Hawthorn fruit extract, Inflammation, Antioxidant, Cholesterol

## Abstract

**Background:**

Trimethylamine-*N*-oxide (TMAO) is an independent risk factor for atherosclerosis. Consumption of hawthorn fruit is believed to be cardio-protective, yet whether it is able to suppress the TMAO-induced atherosclerosis remains unexplored. The present study was to investigate the effects of hawthorn fruit extract (HFE) on TMAO-exacerbated atherogenesis.

**Methods:**

Five groups of male Apolipoprotein E knock-out (ApoE^−/−^) mice were fed a low-fat diet (LFD), a Western high-fat diet (WD), or one of the three WDs containing 0.2% TMAO (WD + TMAO), 0.2% TMAO plus 1% HFE (WD + TMAO + L-HFE), or 0.2% TMAO plus 2% HFE (WD + TMAO + H-HFE), respectively. After 12-weeks of intervention, plasma levels of TMAO, lipid profile, inflammatory biomarkers, and antioxidant enzyme activities were measured. Atherosclerotic lesions in the thoracic aorta and aortic sinus were evaluated. The sterols and fatty acids in the liver and feces were extracted and measured. Hepatic expressions of inflammatory biomarkers and antioxidant enzymes were analyzed.

**Results:**

Dietary TMAO accelerated atherogenesis, exacerbated inflammation, and reduced antioxidant capacities in the plasma and the liver. TMAO promoted hepatic cholesterol accumulation by inhibiting fecal excretion of acidic sterols. HFE could dose-dependently reduce the TMAO-aggravated atherosclerosis and inflammation. HFE was also able to reverse the TMAO-induced reduction in antioxidant capacity by up-regulating the expression of antioxidant enzymes including superoxide dismutase 1 (SOD1), SOD2, glutathione peroxidase 3 (GSH-Px3), and catalase (CAT) in the liver. Moreover, the hepatic cholesterol content was lowered by HFE via enhanced fecal excretion of neutral and acidic sterols.

**Conclusions:**

The present results indicated that HFE was able to reduce the TMAO-exacerbated atherogenesis by attenuating inflammation and improving antioxidant capacity at least in mice.

**Graphic abstract:**

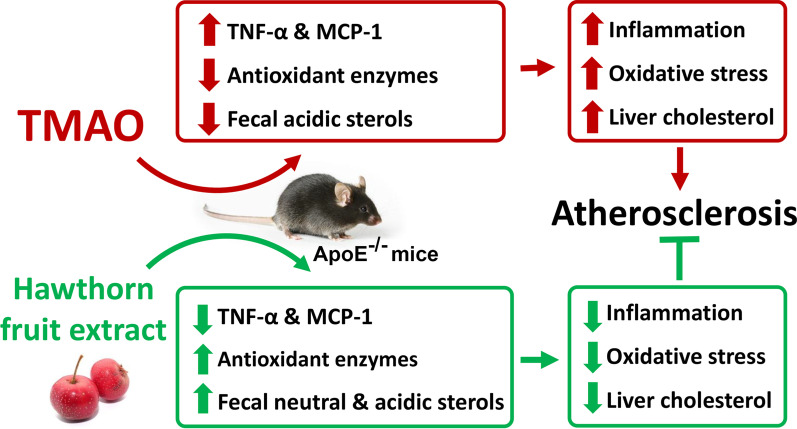

## Introduction

Atherosclerosis is a chronic vascular disease and has become a major underlying cause of cardiovascular deaths worldwide due to its clinical manifestations including myocardial infarction, stroke, and peripheral vascular diseases. It has been well recognized that hypercholesterolemia, vascular inflammation, and oxidative stress are risk factors to initiate and accelerate atherogenesis [1–3]. In the recent decade, high plasma trimethylamine-*N*-oxide (TMAO) has been identified as an independent risk factor for cardiovascular diseases (CVDs) by inducing atherosclerosis [4–6]. In humans, most blood TMAO comes from dietary trimethylamine (TMA)-containing nutrients, for example, phosphatidylcholine, choline, and L-carnitine [4]. After ingestion, these dietary precursors liberate TMA molecule under the actions of bacterial enzymes in the intestine. TMA is then absorbed and oxidized to produce TMAO in the liver by flavin monooxygenases [7].

TMAO promotes atherogenesis mainly by inducing vascular inflammation. It has been reported that TMAO triggers inflammatory gene expression in the aorta of LDLR^−/−^ mice by activating multiple inflammatory signaling pathways including mitogen-activated protein kinase (MAPK), extracellular signal-kinase (ERK), and nuclear factor κB (NF-κB) cascades [8]. The TMAO-triggered formation of NLRP3 inflammasome and subsequent production of interleukin (IL)-1β have been observed in both cultured vascular endothelial cells and mouse aorta [9, 10]. Moreover, TMAO has been shown to promote the formation of foam cells partially mediated by CD36/MAPK/JNK pathway [11]. Disturbance in redox balance by TMAO may also account for its atherogenic activity. It has been demonstrated that TMAO can inhibit the activity of antioxidant enzymes including superoxide dismutase (SOD) and catalase (CAT) in the aorta, leading to a weakened vascular antioxidant defense [10, 12, 13]. Meanwhile, TMAO exacerbates vascular oxidative stress by facilitating the generation of reactive oxygen species (ROS) [10, 12]. Apart from its detrimental effects on the arteries, TMAO has also been shown to disturb cholesterol metabolism and induce liver injury in mice [6, 14–18]. In addition, it has been reported that dietary TMAO aggravates glucose intolerance, obstructs hepatic insulin signaling pathway [19, 20], and cause renal fibrosis and dysfunction in murine models [21].

Hawthorn fruit is the bright red berry of *Crataegus* species. It has been popularly consumed as a fruit and utilized as an herbal medicine for some gastrointestinal disorders in China. Consumption of hawthorn fruit is believed to prevent the development of atherosclerosis with phenolic compounds such as (–)-epicatechin, hyperoside, and isoquercitrin being regarded as the active ingredients [22]. It has been reported that hawthorn ethanolic extract is able to improve vascular function by stimulating endothelium-dependent relaxation in rat isolated mesenteric arteries in vitro [23]. In in vivo studies, administration of hawthorn fruit or its extract can improve hyperlipidemia [24–28], increase the activity of endogenous antioxidant enzymes [29, 30], and reduce inflammation [31]. However, whether hawthorn fruit is able to suppress the TMAO-induced atherosclerosis remains unexplored. Herein, the present study was conducted to investigate if hawthorn fruit extract (HFE) could reduce TMAO-exacerbated atherogenesis in Apolipoprotein E knock-out (ApoE^−/−^) mice fed a Western high-fat diet.

## Methods

### Preparation of HFE

Dried seedless hawthorn fruit were purchased from HK JEBM Ltd. (Hong Kong, China). HFE was prepared according to the method described by Zhang et al. with minor modifications [25]. Briefly, hawthorn fruit was firstly ground into powder. 300 g of hawthorn fruit powder was extracted with one liter of 80% ethanol at room temperature for 24 h for three times. The liquid phase was filtered and pooled, followed by the removal of ethanol in a vacuum rotary evaporator. The aqueous phase was collected and then freeze-dried. The resultant HFE was stored at − 20 °C.

### High performance liquid chromatography (HPLC) analysis of HFE

**Fig. 1:**
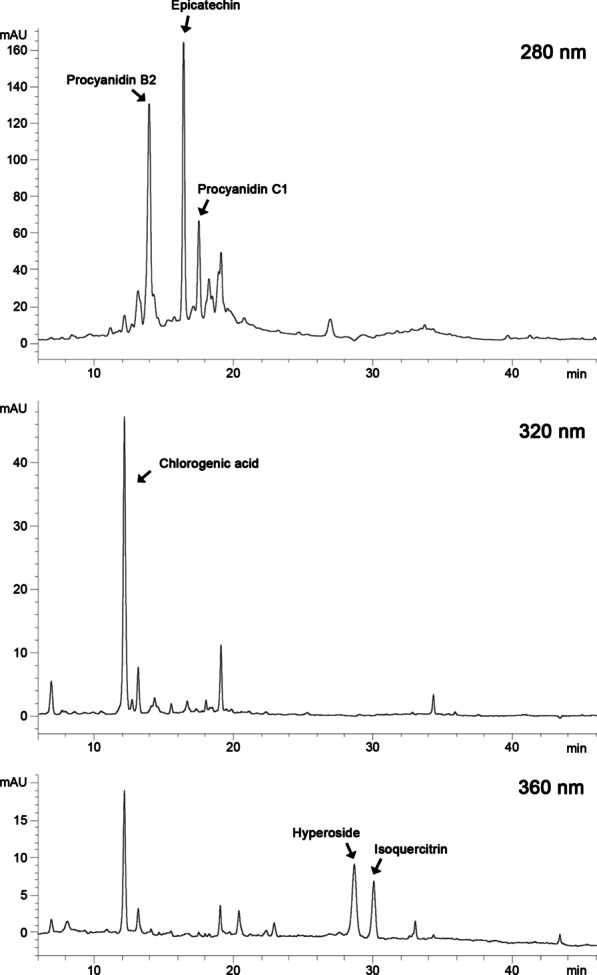
HPLC chromatograms of hawthorn fruit extract at 280, 320, and 360 nm

HPLC analysis was conducted to quantify the phenolic compounds present in HFE based on the method of Liu et al. with minor modifications [32]. Briefly, HFE dissolved in ethanol was filtered, and injected onto a VisionHT C18-HL column (250 × 4.6 mm, 5 μm; Grace, Columbia, MD, USA). The mobile phase consisted of water with 0.5% formic acid (solvent A), and acetonitrile/methanol = 80:20 (v/v) (solvent B), at a flow rate of 1 mL/min. Peaks were monitored at 280, 320, and 360 nm with a UV–visible detector. Phenolic compounds were identified by comparing the retention time with those of authentic standards and quantified according to the calibration curves. The HPLC chromatograms of HFE were shown in Fig. 1. HPLC results showed that 1 g of HFE contained 15.69 mg of total phenolic antioxidant compounds, including 5.93 mg of procyanidin B2, 5.36 mg of epicatechin, 2.45 mg of procyanidin C1, 1.31 mg of chlorogenic acid, 0.36 mg of hyperoside, and 0.28 mg of isoquercitrin.

### Preparation of diets

Five diets were formulated. Briefly, a low-fat diet (LFD) was prepared by mixing all the ingredients listed in Table 1. A Western diet (WD) with 40% energy from fat and 0.15% (w/w) cholesterol was similarly prepared. A WD plus 0.2% TMAO (WD + TMAO) was made by dissolving equimolar TMAO dihydrate (J&K Scientific Ltd., Beijing, China) in distilled water, which was then mixed with the other ingredients. Two experimental diets were prepared by adding 0.2% TMAO plus 1% HFE into WD as a low-dose HFE diet (WD + TMAO + L-HFE), and adding 0.2% TMAO plus 2% HFE into WD as a high-dose HFE diet (WD + TMAO + H-HFE). The diets were then cut into pieces of around 10–15 g cubes and stored at -20 °C until use.

**Table 1:** Composition of five diets^a^

Ingredient (g)	LFD	WD	WD + TMAO	WD + TMAO + L-HFE	WD + TMAO + H-HFE
Corn starch	600.2	414.6	414.6	414.6	414.6
Casein	140.0	167.1	167.1	167.1	167.1
Sucrose	100.0	69.1	69.1	69.1	69.1
Powdered cellulose	50.0	59.7	59.7	59.7	59.7
Lard	40.0	204.3	204.3	204.3	204.3
AIN 76 mineral mix	35.0	41.8	41.8	41.8	41.8
Gelatin	20.0	23.9	23.9	23.9	23.9
AIN 93 vitamin mix	10.0	11.9	11.9	11.9	11.9
Choline bitartrate	2.5	3.0	3.0	3.0	3.0
_DL_-Methionine	2.3	2.7	2.7	2.7	2.7
Cholesterol	–	1.5	1.5	1.5	1.5
TMAO^b^	–	–	2.0	2.0	2.0
Hawthorn fruit extract	–	–	–	10.0	20.0
Total	1000	1000	1002	1012	1022
kcal/g	3.9	4.6	4.6	4.6	4.6
% kcal					
Carbohydrate	73.8	43.1	43.1	43.1	43.1
Protein	16.9	16.9	16.9	16.9	16.9
Fat	9.4	40.0	40.0	40.0	40.0

^a^The five diets include a low-fat diet (LFD), a Western diet (WD) containing 0.15% cholesterol and 40% energy from fat, a WD plus 0.2% TMAO (WD + TMAO), a WD with 0.2% TMAO and 1% hawthorn fruit extract (WD + TMAO + L-HFE), and a WD with 0.2% TMAO and 2% hawthorn fruit extract (WD + TMAO + H-HFE)

^b^TMAO dihydrate equivalent to 0.2% free TMAO was dissolved in distilled water and then mixed with the other ingredients

### Mice

ApoE^−/−^ mice are one of the most commonly used animal models for atherosclerosis studies because they can spontaneously develop hypercholesterolemia and produce atherosclerotic plaque, particularly when they were fed a typical Western high-fat diet [33, 34]. Most importantly, all the phases of atherosclerotic lesions developed in the arterial tree of ApoE^−/−^ mice were very similar to humans [33]. In the present study, forty male ApoE^−/−^ mice (8-week-old) were obtained from the Laboratory Animal Service Center, The Chinese University of Hong Kong. After one-week acclimation, mice were randomly divided into five groups (n = 8 each) and fed one of the five diets for 12 weeks. Food intake were recorded daily, and body weight were recorded weekly. The fecal samples were collected weekly. At the end of week 12, all the mice were sacrificed under isoflurane anesthesia after overnight fasting. Blood and organs were collected and stored at -80 °C until analyses. All the experimental protocols were approved by the Animal Experimental Ethical Committee, The Chinese University of Hong Kong (Ref No. 17/065/MIS).

### Measurement of plasma TMAO

Plasma TMAO were determined as previously described [35]. In brief, 20 μL plasma was mixed with 80 uL methanol for protein precipitation. After centrifugation, the supernatant was collected and subjected to liquid chromatography with tandem mass spectrometry. TMAO was screened in multiple reaction monitoring mode based on the precursor-product ion transitions m/z 76 → 58. Different concentrations of TMAO standard were used to prepare the calibration curve.

### Measurement of plasma lipids and inflammatory biomarkers

Total cholesterol (TC), high-density lipoprotein cholesterol (HDL-C), and triacylglycerol (TG) in the plasma were measured by corresponding commercial enzymatic kits (Stanbio Laboratories, Boerne, TX, USA). Plasma non-HDL-C was determined by deducting HDL-C from TC. Plasma levels of tumor necrosis factor α (TNF-α), IL-1β, IL-6, monocyte chemoattractant protein 1 (MCP-1), and IL-10 were determined by ELISA kits according to the manufacturer’s instructions (CUSABIO Ltd., Wuhan, China).

### Analyses of total antioxidant capacity (T-AOC), antioxidant enzyme activities, and malondialdehyde (MDA) in plasma

Plasma levels of T-AOC, SOD activity, GSH-Px activity, and MDA were determined using the corresponding commercial kits (Nanjing Jiancheng Bioengineering Institute, Nanjing, Jiangsu, China). Plasma T-AOC was determined by reducing Fe^3+^-TPTZ to Fe^2+^-TPTZ under acidic condition. The stable blue color of Fe^2+^-TPTZ was measured at 593 nm. T-AOC was expressed as mM, which was defined as 1 mM of T-AOC had equal antioxidant ability of 1 mM FeSO_4_ standard. The activities of antioxidant enzymes were expressed as a unit per milliliter of plasma (U/mL). The plasma MDA level was expressed as nmol MDA per milliliter of plasma (nmol/mL).

### Evaluation of atherosclerotic lesions

Oil red O staining was used to quantify the atherosclerotic lesions in the thoracic aorta. In brief, the thoracic aorta was carefully cleaned and cut open vertically under microscope, followed by staining in 0.5% oil red O and scanning with a table scanner (Epson 1220 perfection, Epson Co., Tokyo, Japan). The plaque area in the thoracic aorta was quantified by Image J (National Institutes of Health, USA).

For evaluation of atherosclerotic lesions in the aortic sinus, the upper part of heart was fixed, dehydrated, and embedded in paraffin. Serial sections at 5 μm was obtained at aortic sinus using a microtome. The sections were then subjected to routine H&E staining to quantify the area of plaque and necrotic core, and to Masson’s Trichome staining to determine the collagen content in the plaque. Digital micrographs were taken by a Carl Zeiss PALM Inverted microscope (Carl Zeiss, Jena, Germany). Image J was used to quantify the areas of plaque, necrotic core, and collagen.

### Analysis of hepatic cholesterol

Liver cholesterol content was determined using a gas chromatography (GC) method as previously described [36]. Basically, 5α-cholestane was added into liver as an internal standard, followed by extraction with chloroform/methanol (2:1, v/v). After saponification in 1 mol/L NaOH in 90% ethanol, cholesterol was extracted with cyclohexane, converted to its trimethylsilyl (TMS) derivative, and injected onto a SAC-5 capillary column (30 m × 0.25 mm, i.d.; Supelco, Inc., Bellefonte, PA, USA) in a Shimadzu GC-14B equipped with a flame ionization detector for GC analysis.

### Analysis of fecal neutral and acidic sterols

Fecal contents of neutral and acidic sterols were determined using a GC method [35]. In brief, the whole week feces were lyophilized and ground into powder. 5α-Cholestane and hyodeoxycholic acid were added as internal standards for neutral and acidic sterols, respectively. Weighted fecal powder were saponified. The unsaponified fraction was extracted with cyclohexane for neutral sterol analysis, while the saponified aqueous phase was collected for acidic sterol extraction. All the sterols were converted to their corresponding TMS derivatives for GC analysis.

### Measurement of hepatic and fecal lipid contents

Hepatic and fecal lipid contents were quantified by a GC method as previously described [37]. In brief, 100 mg of liver or 300 mg of fecal powder with 0.6 mg of heptadecanoic acid as an internal standard was used for analysis. Total lipid was extracted with chloroform/methanol (2:1, v/v). Fatty acids (FAs) were released and converted to their corresponding fatty acid methyl esters (FAMEs) with 14% boron trifluoride in methanol. The resultant FAMEs were injected onto a HP Innowax capillary column (30 m × 0.32 mm, 0.50 µm film thickness; Agilent Technologies, Santa Clara, CA, USA) for GC analysis.

### Quantitative real-time PCR

Quantitative real-time PCR analyses were conducted as previously described [35]. Basically, RNA was extracted from liver tissue with Trizol (Invitrogen, Carlsbad, CA, USA) and converted to its complementary DNA. Real-time PCR was then performed in a StepOnePlus Real-Time System (Thermo Fisher Scientific, Waltham, MA, USA) using primers shown in Table 2 and SYBR green as a fluorophore. The abundance of mRNA was normalized with that of β-actin.

**Table 2:** Quantitative real-time PCR primer sequences

Gene	Forward primer, 5′ to 3′	Reverse primer, 5′ to 3′
SOD1	AACCAGTTGTGTTGTCAGGAC	CCACCATGTTTCTTAGAGTGAGG
SOD2	CAGACCTGCCTTACGACTATGG	CTCGGTGGCGTTGAGATTGTT
GSH-Px3	CCTTTTAAGCAGTATGCAGGCA	CAAGCCAAATGGCCCAAGTT
CAT	TGGCACACTTTGACAGAGAGC	CCTTTGCCTTGGAGTATCTGG
MCP-1	CCACAACCACCTCAAGCACT	TAAGGCATCACAGTCCGAGTC
IL-6	GAGTGGCTAAGGACCAAGACC	AACGCACTAGGTTTGCCGA
IL-10	GCTCTTACTGACTGGCATGAG	CGCAGCTCTAGGAGCATGTG
IL-1β	GCAACTGTTCCTGAACTCAACT	ATCTTTTGGGGTCCGTCAACT
TNF-α	CCTGTAGCCCACGTCGTAG	GGGAGTAGACAAGGTACAACCC
β-actin	TGTCCACCTTCCAGCAGATGT	AGCTCAGTAACAGTCCGCCTAGA

### Determination of hepatic levels of inflammatory cytokines, T-AOC, antioxidant enzyme activities, and MDA

1.8 mL of phosphate-buffered saline was added into 200 mg of liver tissue, followed by homogenization. After centrifuge, the supernatant was collected and subjected to protein concentration measurement with a Bicinchoninic Acid Protein Assay Kit (Thermo Fisher Scientific, Waltham, MA, USA). Hepatic levels of inflammatory cytokines, T-AOC, activities of antioxidant enzyme, and MDA were measured based on the methods described in 2.6 and 2.7.

### Statistical analysis

All the data were expressed as mean ± SD. One-way ANOVA with post hoc LSD was performed in SPSS software (version 21.0, Chicago, IL, USA) to determine any differences among five groups. Ordinary linear regression was carried out across WD + TMAO, WD + TMAO + L-HFE, and WD + TMAO + H-HFE groups to examine the dose-dependent effect of HFE. Statistical difference was set at *p* < 0.05.

## Results

### Food intake, body weight, and organ weight

After 12-weeks of intervention, results showed that WD feeding raised body weight of mice compared with LFD feeding (Table 3). Addition of TMAO into WD did not affect the body weight. Mice in WD + TMAO + H-HFE group had slightly higher body weight gain compared with those in WD + TMAO group. No difference in daily energy intake or organ weight was found among the five groups of mice (Table 3).

**Table 3:** Food intake, body weight, and organ weight in ApoE^−/−^ mice

	LFD	WD	WD + TMAO	WD + TMAO + L-HFE	WD + TMAO + H-HFE	*p* value
Food intake						
Daily food intake (g/d)	3.96 ± 0.23^a^	3.32 ± 0.10^b^	3.16 ± 0.10^b^	3.20 ± 0.15^b^	3.29 ± 0.29^b^	0.002
Daily energy intake (kcal/d)	15.25 ± 0.89	15.28 ± 0.46	14.51 ± 0.47	14.54 ± 0.67	14.81 ± 1.31	0.662
Body weight (g)						
Initial	24.8 ± 1.4	24.8 ± 1.1	25.0 ± 1.0	25.2 ± 0.8	24.8 ± 0.8	0.950
Final	28.1 ± 1.4^c^	30.0 ± 1.1^ab^	29.1 ± 1.2^bc^	30.2 ± 1.4^ab^	30.7 ± 1.6^a^	0.006
Organ weight (% body weight)						
Liver	4.00 ± 0.22	3.84 ± 0.06	3.86 ± 0.14	3.89 ± 0.08	3.85 ± 0.11	0.133
Testis	0.61 ± 0.10	0.62 ± 0.05	0.69 ± 0.06	0.63 ± 0.12	0.57 ± 0.12	0.202
Kidney	1.05 ± 0.08	1.00 ± 0.07	1.02 ± 0.05	1.05 ± 0.09	1.03 ± 0.05	0.544
Epididymal adipose tissue	2.21 ± 0.37	1.77 ± 0.61	1.91 ± 0.54	2.03 ± 0.33	2.15 ± 0.74	0.513
Perirenal adipose tissue	0.77 ± 0.26	0.59 ± 0.28	0.66 ± 0.25	0.67 ± 0.23	0.87 ± 0.31	0.317
Heart	0.64 ± 0.05	0.63 ± 0.09	0.70 ± 0.07	0.67 ± 0.06	0.69 ± 0.06	0.231

Mice were fed a low-fat diet (LFD), a Western diet (WD), or one of the three WDs containing 0.2% TMAO (WD + TMAO), 0.2% TMAO plus 1% hawthorn fruit extract (WD + TMAO + L-HFE), or 0.2% TMAO plus 2% hawthorn fruit extract (WD + TMAO + H-HFE) for 12 weeks. Data are expressed as mean ± SD, n = 8. ^a,b,c^means in the same row with different letters differ significantly at *p* < 0.05

### Plasma lipid profiles

After 12-week intervention, WD feeding disturbed plasma lipid profiles in mice by raising plasma levels of TC, TG, and non-HDL-C, and reducing plasma HDL-C concentration compared with LFD feeding (Table 4). Neither TMAO nor HFE significantly altered plasma lipids in WD-fed mice. Compared to feeding WD + TMAO diet, high-dose HFE supplementation tended to decrease plasma TC (*p* = 0.101) but this decrement did not reach statistical significance.

**Table 4:** Plasma lipids in ApoE^−/−^ mice

	LFD	WD	WD + TMAO	WD + TMAO + L-HFE	WD + TMAO + H-HFE	*p* value	*p* for dose effect
TC (mg/dL)	673 ± 73^b^	1163 ± 156^a^	1250 ± 134^a^	1177 ± 153^a^	1138 ± 133^a^	< 0.001	0.117
HDL-C (mg/dL)	74 ± 20^a^	46 ± 15^b^	46 ± 20^b^	41 ± 19^b^	43 ± 13^b^	0.016	0.745
TG (mg/dL)	131 ± 22^b^	216 ± 43^a^	226 ± 28^a^	255 ± 48^a^	224 ± 42^a^	< 0.001	0.143
Non-HDL-C (mg/dL)	600 ± 67^b^	1117 ± 154^a^	1204 ± 152^a^	1136 ± 161^a^	1095 ± 130^a^	< 0.001	0.919
Non-HDL-C/HDL-C	8.64 ± 2.27^b^	27.06 ± 11.32^a^	31.99 ± 15.98^a^	31.92 ± 12.74^a^	27.30 ± 8.81^a^	0.006	0.464
HDL-C/TC	0.11 ± 0.03^a^	0.04 ± 0.01^b^	0.04 ± 0.02^b^	0.04 ± 0.02^b^	0.04 ± 0.01^b^	< 0.001	0.982

Mice were fed a low-fat diet (LFD), a Western diet (WD), or one of the three WDs containing 0.2% TMAO (WD + TMAO), 0.2% TMAO plus 1% hawthorn fruit extract (WD + TMAO + L-HFE), or 0.2% TMAO plus 2% hawthorn fruit extract (WD + TMAO + H-HFE) for 12 weeks. Data are expressed as mean ± SD, n = 8

TC, total cholesterol; HDL-C, high-density lipoprotein cholesterol; TG, triacylglycerol; Non-HDL-C, non-HDL cholesterol

^a,b^Means in the same row with different letters differ significantly at *p* < 0.05 by one-way ANOVA. *p* for dose effect was analyzed using ordinary linear regression across WD + TMAO, WD + TMAO + L-HFE, and WD + TMAO + H-HFE groups

### Plasma TMAO level

**Fig. 2:**
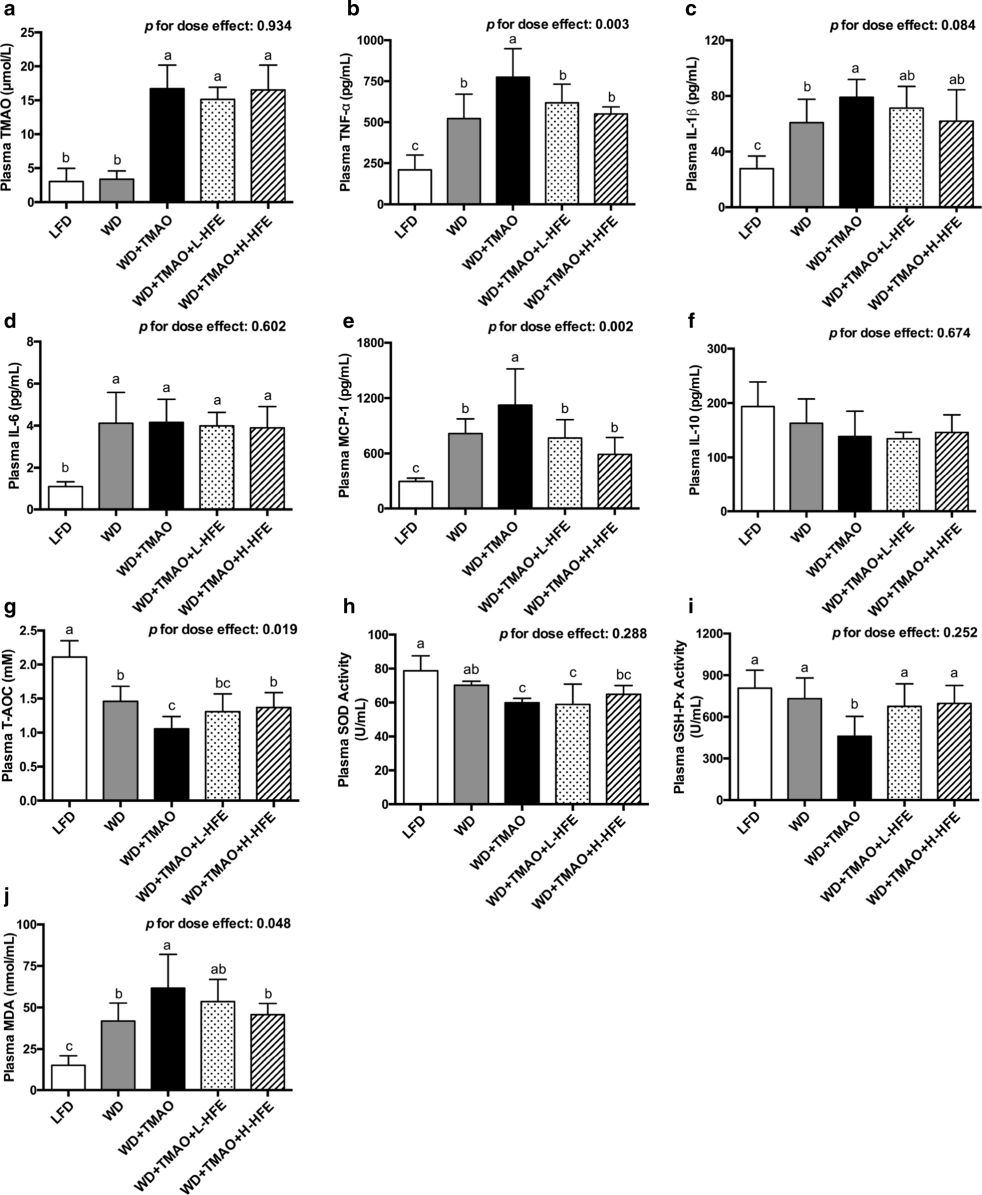
Plasma levels of TMAO, inflammatory cytokines, and oxidative stress-related parameters in ApoE^−/−^ mice. **a** Plasma level of TMAO. The inflammatory cytokines include **b** tumor necrosis factor α (TNF-α), **c** interleukin (IL)-1β, **d** IL-6, **e** monocyte chemoattractant protein 1 (MCP-1), and **f** IL-10. Plasma levels of **g** total antioxidant capacity (T-AOC), **h** superoxide dismutase (SOD) activity, **i** glutathione peroxidase (GSH-Px) activity, and **j** malondialdehyde (MDA) in ApoE^−/−^ mice. Data are expressed as mean with SD, n = 8. ^a,b,c^means with different superscript letters differ significantly at *p* < 0.05 by one-way ANOVA. *p* for dose effect was analyzed using ordinary linear regression across WD + TMAO, WD + TMAO + L-HFE, and WD + TMAO + H-HFE groups

Similar plasma TMAO level was observed in mice maintained on LFD and WD (Fig. 2a). Addition of 0.2% TMAO into WD increased plasma TMAO level in mice by four folds compared with feeding of diets without TMAO. HFE supplementation did not affect plasma TMAO concentration in mice (Fig. 2a), suggesting that the absorption of dietary TMAO was not altered by HFE administration.

### Plasma inflammatory biomarkers

Results on plasma inflammatory biomarkers revealed that WD feeding raised plasma levels of pro-inflammatory cytokines including TNF-α, IL-1β, IL-6, and MCP-1 compared with LFD feeding (Fig. 2b–e). Addition of TMAO into WD aggravated inflammation by further elevating TNF-α, IL-1β, and MCP-1 levels in the plasma (Fig. 2b, c, e). Compared with WD + TMAO diet, addition of HFE reduced plasma concentrations of TNF-α and MCP-1 in a dose-dependent manner (Fig. 2b, e). No significant difference in plasma IL-10 level was observed among the five groups of mice (Fig. 2f).

### Plasma T-AOC, antioxidant enzyme activities, and MDA

Mice maintained on WD had lower plasma T-AOC and higher plasma level of MDA, a marker of lipid peroxidation [38], compared with those on LFD (Fig. 2g, j). Addition of TMAO into WD exacerbated oxidative stress by reducing plasma T-AOC, decreasing the activity of antioxidant enzymes SOD and GSH-Px, and elevating plasma MDA concentration (Fig. 2g–j). The TMAO-induced reductions in plasma T-AOC and GSH-Px activity were restored by HFE administration (Fig. 2g, i). Moreover, it was observed that HFE dose-dependently reduced plasma MDA level against TMAO in mice (Fig. 2j).

### Atherosclerotic lesions

**Fig. 3:**
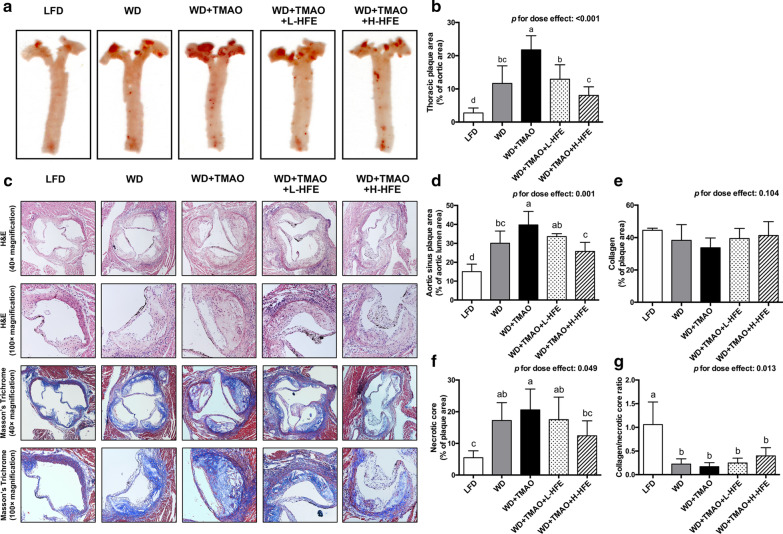
Atherosclerotic lesions in ApoE^−/−^ mice. **a** Representative images and **b** quantification of en face oil-red-O stained plaque area in the thoracic aorta. **c** Representative images of cross-sections at the aortic sinus stained with H&E and Masson’s Trichrome. Images were taken at 40 × and 100 × magnification. Quantification of **d** plaque area, **e** collagen content, **f** necrotic core area, and **g** the ratio of collagen to necrotic core in the aortic sinus of ApoE^−/−^ mice. Data are expressed as mean with SD, n = 8. ^a,b,c,d^means with different superscript letters differ significantly at *p* < 0.05 by one-way ANOVA. *p* for dose effect was analyzed using ordinary linear regression across WD + TMAO, WD + TMAO + L-HFE, and WD + TMAO + H-HFE groups

WD feeding promoted the formation of plaque in mouse aorta (Fig. 3a–g). Compared with those in WD group, mice in WD + TMAO group had plaque area increased by 87% in thoracic aorta and by 32% in aortic sinus, respectively (Fig. 3a–d). Addition of HFE was able to diminish plaque development by both longitudinal and cross-sectional evaluations, and inhibit the formation of necrotic core in aortic sinus in a dose-dependent fashion (Fig. 3a–d, f). However, no significant difference in collagen content or the ratio of collagen to necrotic core, an indicator for plaque stability, was found among the four groups of mice fed high-fat WDs.

### Hepatic cholesterol and lipid contents

**Fig. 4:**
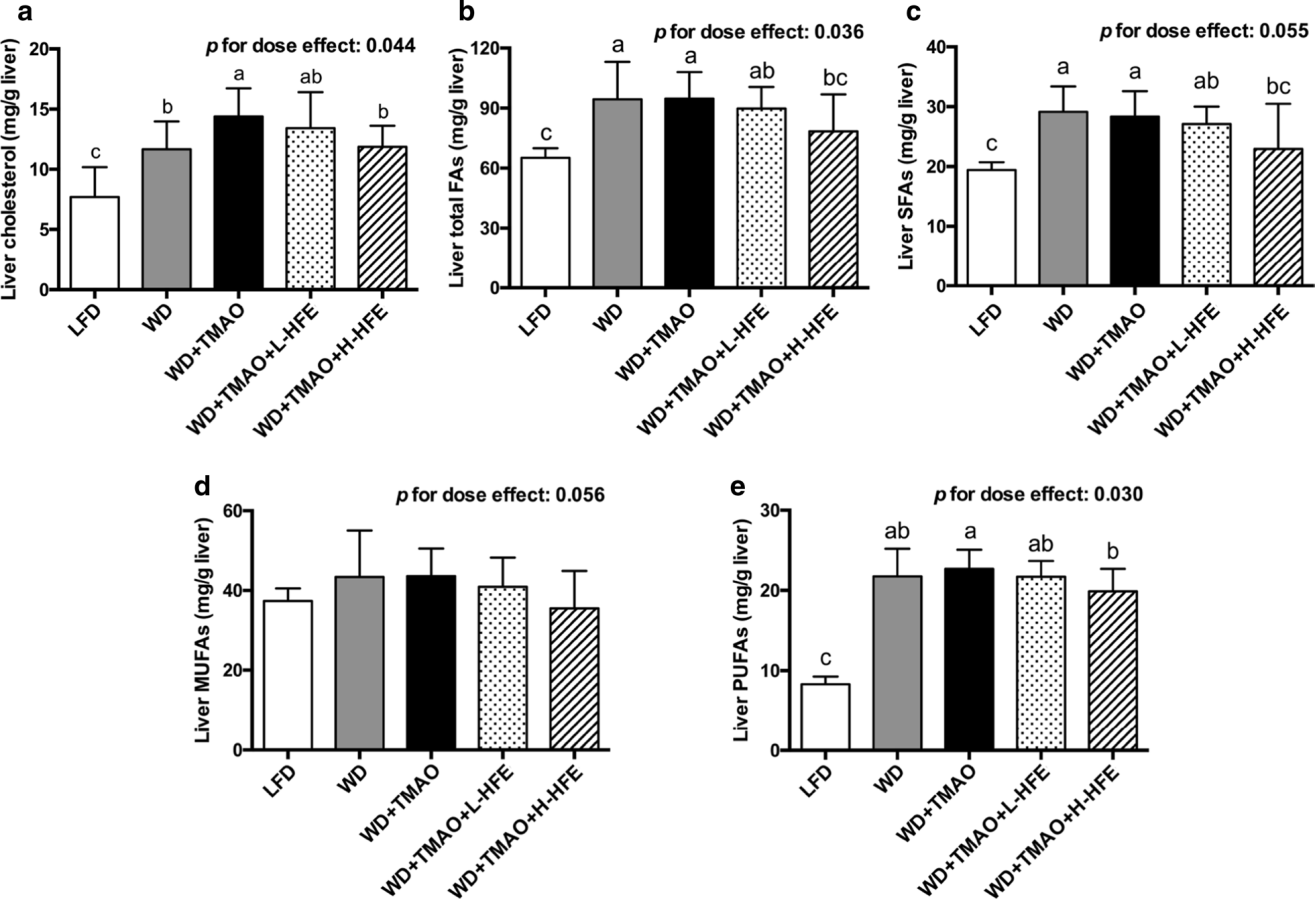
Changes in hepatic lipid contents in ApoE^−/−^ mice. Hepatic contents of **a** cholesterol, **b** total fatty acids (FAs), **c** saturated fatty acids (SFAs), **d** monounsaturated fatty acids (MUFAs), and **e** polyunsaturated fatty acids (PUFAs) in ApoE^−/−^ mice. Data are expressed as mean with SD, n = 8. ^a,b,c^means with different superscript letters differ significantly at *p* < 0.05 by one-way ANOVA. *p* for dose effect was analyzed using ordinary linear regression across WD + TMAO, WD + TMAO + L-HFE, and WD + TMAO + H-HFE groups

Compared with LFD feeding, WD feeding increased hepatic cholesterol content and the accumulation of lipids in the liver (Fig. 4a–e). Addition of TMAO promoted the liver cholesterol accumulation but it did not alter the hepatic levels of FAs (Fig. 4a–e). Compared with WD + TMAO diet, HFE supplementation decreased hepatic cholesterol level in a dose-dependent manner (Fig. 4a). In addition, the hepatic accumulation of total FAs, saturated fatty acids (SFAs), and polyunsaturated fatty acids (PUFAs) was significantly reduced in WD + TMAO + H-HFE group (Fig. 4b, c, e).

### Fecal sterols and lipids

Results showed that coprostanol, coprostanone, cholesterol, and dihydrocholsterol were the predominant neutral sterols in mouse feces (Table 5). Mouse fecal acidic sterols mainly consisted of lithocholic acid, deoxycholic acid, chenodeoxycholic acid, and cholic acid. WD feeding strikingly promoted the excretion of total neutral and acidic sterols in feces (Table 5). Addition of TMAO into WD did not influence the fecal neutral sterol contents, but it significantly decreased the fecal output of total acidic sterols by 41%. Compared with those on WD + TMAO diet, mice on WD + TMAO + L-HFE and WD + TMAO + H-HFE diets had fecal contents of total neutral sterols increased by 68% and 100%, respectively. Moreover, the TMAO-induced reduction in fecal output of acidic sterols was reversed by HFE supplementation in a dose-dependent manner (Table 5).

**Table 5:** Fecal contents of sterols and lipids at week 12 in ApoE^−/−^ mice

	LFD	WD	WD + TMAO	WD + TMAO + L-HFE	WD + TMAO + H-HFE	*p* value	*p* for dose effect
Week 12 fecal neutral sterols (mg/d)							
Coprostanol	0.01 ± 0.00^c^	0.39 ± 0.07^b^	0.44 ± 0.15^b^	0.70 ± 0.21^a^	0.61 ± 0.12^a^	< 0.001	0.123
Coprostanone	0.01 ± 0.01	0.02 ± 0.02	0.09 ± 0.10	0.16 ± 0.16	0.10 ± 0.13	0.105	0.921
Cholesterol	0.09 ± 0.03^c^	0.53 ± 0.25^b^	0.38 ± 0.32^bc^	0.69 ± 0.35^b^	1.13 ± 0.56^a^	< 0.001	0.006
Dihydrocholesterol	0.02 ± 0.01^c^	0.05 ± 0.01^ab^	0.04 ± 0.02^b^	0.05 ± 0.02^ab^	0.06 ± 0.02^a^	0.001	0.064
Total neutral sterols	0.12 ± 0.03^c^	0.99 ± 0.19^b^	0.95 ± 0.28^b^	1.60 ± 0.41^a^	1.90 ± 0.41^a^	< 0.001	< 0.001
Week 12 fecal acidic sterols (mg/d)							
Lithocholic acid	0.24 ± 0.02^c^	0.74 ± 0.24^bc^	0.66 ± 0.21^bc^	1.25 ± 0.34^ab^	1.61 ± 0.55^a^	0.003	0.016
Deoxycholic acid	0.40 ± 0.06^b^	0.80 ± 0.35^a^	0.46 ± 0.12^b^	0.59 ± 0.05^ab^	0.57 ± 0.01^ab^	0.101	0.119
Chenodeoxycholic acid	0.17 ± 0.04^c^	0.54 ± 0.16^a^	0.30 ± 0.07^bc^	0.40 ± 0.03^ab^	0.31 ± 0.03^bc^	0.003	0.807
Cholic acid	0.31 ± 0.12^b^	0.97 ± 0.21^a^	0.39 ± 0.15^b^	0.51 ± 0.28^b^	0.45 ± 0.05^b^	0.008	0.693
Total acidic sterols	1.11 ± 0.22^c^	3.05 ± 0.84^a^	1.81 ± 0.47^bc^	2.75 ± 0.36^ab^	2.94 ± 0.62^a^	0.006	0.028
Week 12 fecal lipids (mg/d)							
Total SFAs	0.59 ± 0.08^b^	7.04 ± 1.02^a^	7.08 ± 0.46^a^	6.76 ± 1.76^a^	8.20 ± 2.55^a^	< 0.001	0.471
Total MUFAs	0.21 ± 0.03^b^	0.78 ± 0.11^a^	0.76 ± 0.05^a^	0.84 ± 0.24^a^	1.04 ± 0.49^a^	< 0.001	0.162
Total PUFAs	0.10 ± 0.01^c^	0.20 ± 0.03^b^	0.19 ± 0.01^b^	0.21 ± 0.04^b^	0.28 ± 0.10^a^	< 0.001	0.176
Total FAs	0.90 ± 0.12^b^	8.02 ± 1.15^a^	8.04 ± 0.51^a^	7.81 ± 2.03^a^	9.52 ± 3.13^a^	< 0.001	0.411

Mice were fed a low-fat diet (LFD), a Western diet (WD), or one of the three WDs containing 0.2% TMAO (WD + TMAO), 0.2% TMAO plus 1% hawthorn fruit extract (WD + TMAO + L-HFE), or 0.2% TMAO plus 2% hawthorn fruit extract (WD + TMAO + H-HFE) for 12 weeks. Data are expressed as mean ± SD, n = 8

SFA, saturated fatty acid; MUFA, monounsaturated fatty acid; PUFA, polyunsaturated fatty acid; FA, fatty acid

^a,b,c^Means in the same row with different letters differ significantly at *p* < 0.05 by one-way ANOVA. *p* for dose effect was analyzed using ordinary linear regression across WD + TMAO, WD + TMAO + L-HFE, and WD + TMAO + H-HFE groups

For fecal FA analysis, a ninefold increment in fecal total FA content was observed in WD-fed mice compared with those in LFD-fed mice (Table 5). Addition of TMAO into WD did not alter the fecal output of FAs. Compared with those on WD + TMAO diet, mice on WD + TMAO + H-HFE diet had higher fecal content of PUFAs. However, HFE administration did not significantly affect the fecal excretion of SFAs, MUFAs, or total FAs (Table 5).

### Hepatic gene expression of inflammatory cytokines

**Fig. 5:**
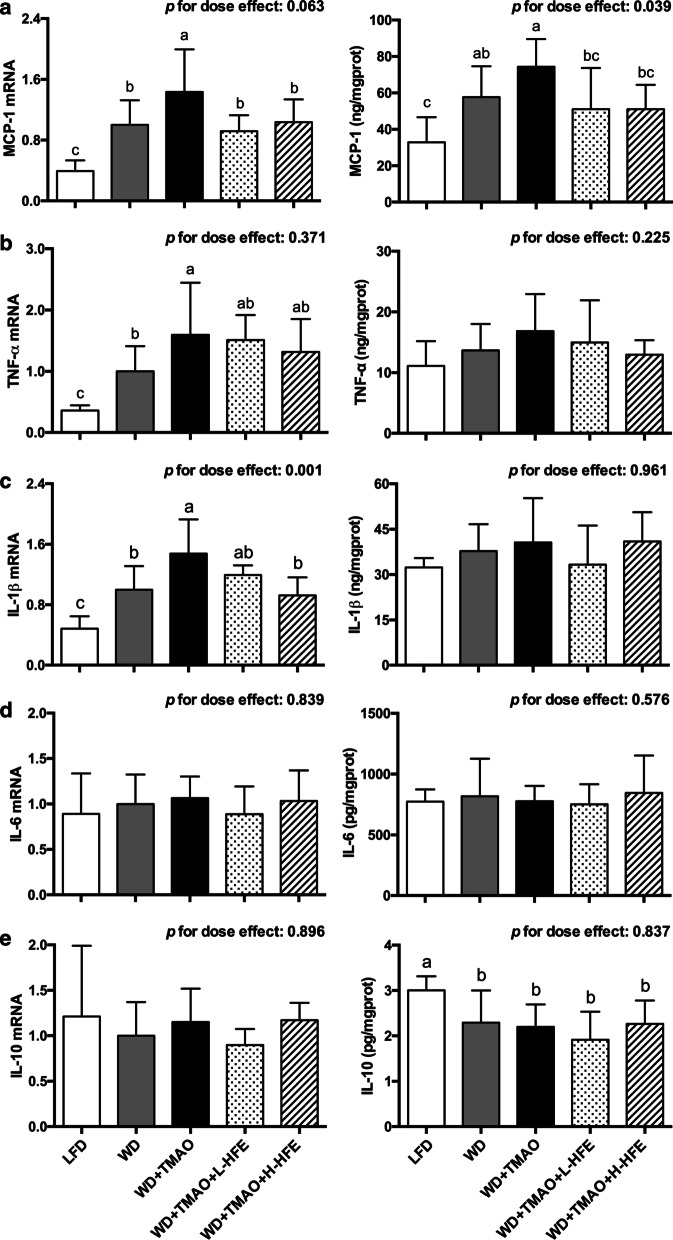
Relative expression of inflammatory cytokines in the liver of ApoE^−/−^ mice. The mRNA and protein levels of **a** monocyte chemoattractant protein 1 (MCP-1), **b** tumor necrosis factor α (TNF-α), **c** interleukin (IL)-1β, **d** IL-6, and **e** IL-10 in the liver of ApoE^−/−^ mice. Data are expressed as mean with SD, n = 8. ^a,b,c^means with different superscript letters differ significantly at *p* < 0.05 by one-way ANOVA. *p* for dose effect was analyzed using ordinary linear regression across WD + TMAO, WD + TMAO + L-HFE, and WD + TMAO + H-HFE groups

WD feeding stimulated hepatic inflammation by facilitating the expression of MCP-1 at both transcriptional and translational levels, and increasing the mRNA level of TNF-α and IL-1β (Fig. 5a–c). Addition of TMAO into WD further exacerbated inflammation in the liver by up-regulating the mRNA expression of MCP-1, TNF-α, and IL-1β (Fig. 5a–c). Compared with feeding WD + TMAO diet, feeding WD + TMAO + L-HFE and WD + TMAO + H-HFE diets significantly lowered the mRNA and protein abundance of MCP-1 in the liver (Fig. 5a). Besides, the mRNA expression of IL-1β was dose-dependently down-regulated by dietary HFE (Fig. 5c). Neither TMAO nor HFE significantly affected the mRNA or protein levels of IL-6 and IL-10 in the liver (Fig. 5d, e).

### Hepatic gene expression of antioxidant enzymes and their activities

**Fig. 6:**
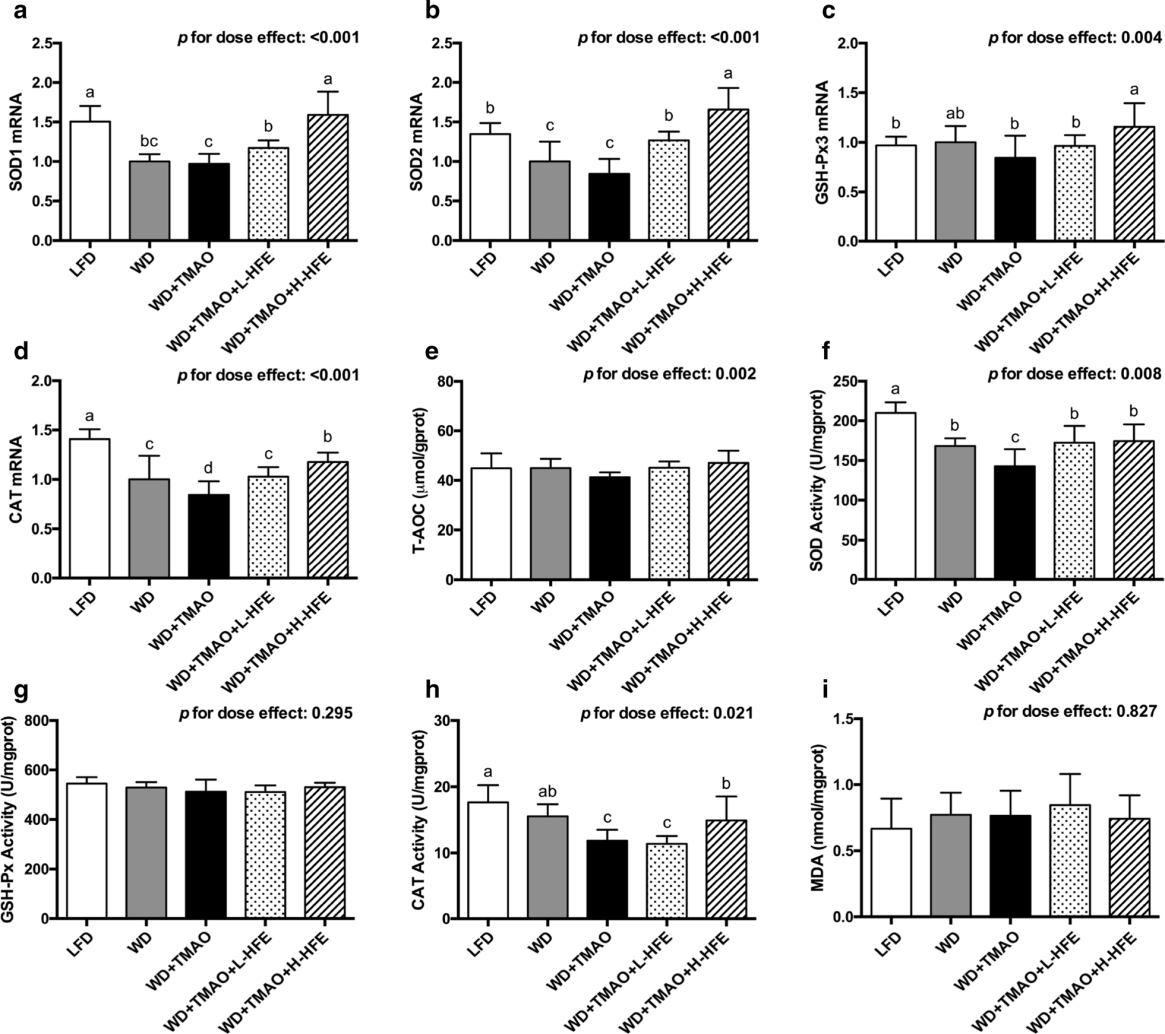
The expression and activity of antioxidant enzyme in the liver of ApoE^−/−^ mice. The mRNA levels of **a** superoxide dismutase 1 (SOD1), **b** SOD2, **c** glutathione peroxidase 3 (GSH-Px3), and **d** catalase (CAT) in mice. **e** Hepatic total antioxidant capacity (T-AOC) in mice. Activity of **f** SOD, **g** GSH-Px, and **h** CAT in the liver of mice. **i** Hepatic malondialdehyde (MDA) level in mice. Data are expressed as mean with SD, n = 8. ^a,b,c,d^means with different superscript letters differ significantly at *p* < 0.05 by one-way ANOVA. *p* for dose effect was analyzed using ordinary linear regression across WD + TMAO, WD + TMAO + L-HFE, and WD + TMAO + H-HFE groups

The hepatic expression of several antioxidant enzymes was then examined. SOD 1 and SOD 2 genes encode the cytoplasmic Cu/Zn-SOD and mitochondrial Mn-SOD, respectively [39]. GSH-Px3 is responsible for the major GSH-Px activity in plasma [40]. CAT catalyzes the decomposition of hydrogen peroxide [41]. After 12-week intervention, WD feeding lowered the antioxidant defense capacity in the liver by down-regulating the mRNA expression of SOD1, SOD2, and CAT, and reducing the activity of SOD (Fig. 6a, b, d, f). Compared with those on WD diet, a significant decrement in the mRNA level of CAT, and lowered activity of SOD and CAT were observed in mice maintained on WD + TMAO diet (Fig. 6d, f, h). The mRNA abundance of SOD1, SOD2, GSH-Px3, and CAT was dose-dependently raised by dietary HFE (Fig. 6a–d). Furthermore, the TMAO-induced reduction in SOD activity was reversed by both low-dose and high-dose HFE, while the TMAO-reduced CAT activity was improved in WD + TMAO + H-HFE group (Fig. 6f, h). However, no obvious difference in hepatic T-AOC, GSH-Px activity, or MDA level were observed among the five groups of mice (Fig. 6e, g, i).

## Discussion

Recent studies have shown that TMAO is positively correlated with the risk of adverse CVDs in humans [4, 5]. Hawthorn fruit consumption is considered beneficial to cardiovascular health. To the best of our knowledge, the present study was the first of its kinds to investigate the effect of HFE on TMAO-exacerbated atherogenesis in mice. The present results clearly demonstrated that TMAO accelerated atherogenesis, exacerbated inflammation, and aggravated oxidative stress in ApoE^−/−^ mice fed a high-fat WD. Consistent with the previous report [29], the present results demonstrated that HFE was able to inhibit the formation of atherosclerotic plaque in mice fed a TMAO-containing diet.

Atherosclerosis is a progressive inflammatory vascular disease [2]. Activated vascular inflammation can trigger endothelial dysfunction, promote the formation of foam cells, and accelerate atherosclerosis development [42]. Amongst various inflammatory cytokines and chemokines, the production of MCP-1 by aortic endothelial cells is markedly enhanced at the initiation stage of atherosclerosis [43, 44]. MCP-1 functions to recruit monocytes and facilitate their infiltration into the vascular intima [44]. Overexpression of MCP-1 could accelerate atherogenesis in ApoE^−/−^ mice by promoting the accumulation of macrophages and oxidized lipids in the aorta [43]. TNF-α exerts a potent pro-atherogenic activity by up-regulating the expression of adhesion molecules and MCP-1, increasing the permeability of endothelium, and stimulating the activation of macrophages [45]. IL-1β and IL-6 are the predominant pro-inflammatory ILs in atherosclerosis mainly by aggravating subsequent inflammatory cascades, mediating the acute-phase response, and promoting the fatty streak formation in atherogenesis [46]. IL-10 is generally considered as an anti-atherogenic cytokine due to its anti-inflammatory properties [47]. Our results clearly showed that dietary TMAO increased plasma levels of TNF-α, IL-1β, and MCP-1 in mice fed a high-fat WD (Fig. 2b, c, e). Consistently, the hepatic mRNA expression of these three proinflammatory cytokines were up-regulated by TMAO (Fig. 5a–c). Together with the findings that dietary TMAO elevated serum concentrations of MCP-1 and IL-1β, and induced inflammation in adipose tissue of high-fat-fed mice [19, 20], our results demonstrated that TMAO was pro-atherogenic and aggravated extensive inflammation. In the present study, HFE supplement significantly lowered plasma levels of TNF-α and MCP-1 in a dose-dependent manner (Fig. 2b, e) and down-regulated the hepatic expression of MCP-1 and IL-1β (Fig. 5a, c). Our data were in agreement with the study of Zhang et al., who reported that the sugar-free aqueous extract of hawthorn fruit lowered serum levels of inflammatory biomarkers including IL-1β, IL-8, IL-18, and C-reactive protein (CRP) in rats fed a high-fat diet [31]. The present results suggested that HFE supplement could alleviate TMAO-exacerbated inflammation.

Oxidative stress is a redox imbalance with increased production of ROS and overwhelmed antioxidant defense capacity [48]. Endogenous antioxidant enzymes including SOD, GSH-Px, and CAT are critical for the maintenance of redox balance by scavenging excessive ROS [41]. Severe oxidative stress can lead to endothelial dysfunction, LDL oxidative modification, and atherogenesis [48]. Previous studies showed that TMAO stimulated ROS production and inhibited the SOD activation both in human umbilical vein endothelial cells (HUVEC) and rodent animals [10, 12, 13]. Similarly, the present results demonstrated that dietary TMAO lowered plasma T-AOC, reduced the activities of SOD and GSH-Px in the plasma, and increased the plasma level of MDA (Fig. 2g–j). The hepatic activity of SOD and CAT were also decreased in mice exposed to TMAO-containing diet (Fig. 6f, h). The present data suggested that dietary TMAO exacerbated oxidative stress and weakened the antioxidant defenses in mice.

The cardio-protective effect of HFE was partially attributed to its potent antioxidant capacity. Previous study showed that seven antioxidant phenolic compounds (epicatechin, chlorogenic acid, hyperoside, isoquecitrin, protocatechuic acid, rutin, and quercetin) isolated from the ethyl acetate fraction of HFE were able to prevent the Cu^2+^-mediated oxidation of human low-density lipoprotein (LDL) and the peroxy free radical-induced oxidation of α-tocopherol in human LDL in vitro [22]. In the present study, HFE containing 15.69 mg/g total phenolic antioxidants conspicuously enhanced the antioxidant defenses and relieved the TMAO-exacerbated oxidative stress both in the plasma and the liver (Figs. 2g–j, 6f, h). At a molecular level, the hepatic mRNA expression of SOD1, SOD2, GSH-Px3, and CAT was up-regulated by HFE in a dose-dependent manner (Fig. 6a–d). Together with the finding that HFE could improve the expression of antioxidant enzymes in the serum, liver, and brain of senescence-accelerated mice [30], our results clearly demonstrated that HFE possessed antioxidant capacity in both the plasma and the liver, thereby contributing to its anti-atherogenic activity against TMAO in mice.

Disturbed cholesterol metabolism may lead to cholesterol accumulation and the development of atherosclerosis [49, 50]. In the present study, TMAO administration elevated hepatic cholesterol concentration without affecting FA content in the liver (Fig. 4a–e). Fecal excretions of neutral and acidic sterols are the major routes for cholesterol elimination [50]. Consistent with the report of Chen et al. [14] and our previous studies [35, 51], the present results showed that the TMAO-induced hepatic cholesterol accumulation was probably mediated by decreasing the fecal output of acidic sterols (Table 5). However, no significant alterations in plasma TC, HDL-C, or non-HDL-C was observed in mice fed dietary TMAO (Table 4), which was in line with the findings that 0.12% dietary TMAO induced atherogenesis but it did not change the plasma TC in ApoE^−/−^ mice [4, 6]. In contrast, some researchers observed an increment in plasma TC in mice given diets containing high content of TMAO or choline, a dietary precursor of TMAO [14, 16, 17, 35]. Contradictorily, it was reported that 0.2% dietary TMAO led to decreased plasma TC and TG levels in high-fat-fed C57BL/6 J mice [19]. The inconclusive effect of TMAO on plasma cholesterol may be affected by other dietary components, as well as the rodent models adopted.

In the present study, we observed that HFE administration could dose-dependently decrease the hepatic cholesterol content via enhancement on the fecal excretion of neutral and acidic sterols (Fig. 4a and Table 5), but it did not significantly modulate plasma lipid profiles in ApoE^−/−^ mice exposed to dietary TMAO (Table 4). The inconsistent effect of TMAO or HFE on plasma cholesterol and liver cholesterol might be explained by the complete deletion of ApoE gene in ApoE^−/−^ mice. Wild-type mice are generally resistant to atherosclerosis, because most of their cholesterol is transported in HDL particles, but not in non-HDL particles as in humans [34]. ApoE, a 34 kDa glycoprotein, is a structural component of lipoprotein particles, and functions as a recognition factor for the clearance of chylomicrons and very low-density lipoprotein (VLDL) remnants from circulation by the liver [52]. Genetic manipulation by knocking out ApoE gene in mice results in spontaneous development of hypercholesterolemia (dominated by non-HDL-C) and atherosclerosis [34]. Moreover, similar lesion distribution and progression as those in humans could be observed in ApoE^−/−^ mice [33, 53], making them a particularly popular animal model in atherosclerosis studies. Study showed that compared to their wild-type counterparts, ApoE^−/−^ mice had 10 times higher plasma cholesterol level, whereas their liver cholesterol content was less affected and only increased by 56% [54]. Therefore, the absence of ApoE impedes the exchange between plasma cholesterol and hepatic cholesterol, and might alter the impact of TMAO or HFE on plasma cholesterol regulation.

## Conclusion

In summary, the present study demonstrated that TMAO accelerated atherosclerosis development by inducing extensive inflammation and oxidative stress in high-fat-fed ApoE^−/−^ mice. Moreover, TMAO disturbed cholesterol metabolism by inhibiting fecal acidic sterol excretion, resulting in elevated hepatic cholesterol accumulation. Phenolic antioxidants-enriched HFE could reduce atherogenesis, alleviate inflammation, improve antioxidant defense capacity, and partially reverse the TMAO-induced elevation in hepatic cholesterol content in mice given dietary TMAO. It was therefore concluded that HFE was protective against the TMAO-exacerbated atherogenesis in ApoE^−/−^ mice mainly via its anti-inflammatory and antioxidant activities.

## Data Availability

The datasets used and/or analysed during the current study are available from the corresponding author on reasonable request.

## Abbreviations

ApoE: Apolipoprotein E
CAT: Catalase
CVD: Cardiovascular disease
ERK: Extracellular signal-kinase
FA: Fatty acid
FAME: Fatty acid methyl ester
GC: Gas chromatography
GSH-Px3: Glutathione peroxidase 3
HDL-C: High-density lipoprotein cholesterol
HFE: Hawthorn fruit extract
HPLC: High performance liquid chromatography
IL: Interleukin
LFD: Low-fat diet
MAPK: Mitogen-activated protein kinase
MCP-1: Monocyte chemoattractant protein 1
MDA: Malondialdehyde
MUFA: Monounsaturated fatty acid
NF-κB: Nuclear factor κB
PUFA: Polyunsaturated fatty acid
ROS: Reactive oxygen species
SFA: Saturated fatty acid
SOD1: Superoxide dismutase 1
SOD2: Superoxide dismutase 2
T-AOC: Total antioxidant capacity
TC: Total cholesterol
TG: Triacylglycerol
TMA: Trimethylamine
TMAO: Trimethylamine-N-oxide
TMS: Trimethylsilyl
TNF-α: Tumor necrosis factor α
WD: Western high-fat diet
